# Can variation in standard metabolic rate explain context‐dependent performance of farmed Atlantic salmon offspring?

**DOI:** 10.1002/ece3.4716

**Published:** 2018-12-26

**Authors:** Grethe Robertsen, Donald Reid, Sigurd Einum, Tonje Aronsen, Ian A. Fleming, Line E. Sundt‐Hansen, Sten Karlsson, Eli Kvingedal, Ola Ugedal, Kjetil Hindar

**Affiliations:** ^1^ Norwegian Institute for Nature Research Trondheim Norway; ^2^ School of Life Sciences, College of Medical, Veterinary and Life Sciences University of Glasgow Glasgow UK; ^3^ Centre for Biodiversity Dynamics, Department of Biology Norwegian University of Science and Technology Trondheim Norway; ^4^ Department of Ocean Sciences Memorial University of Newfoundland St John’s Newfoundland Canada

**Keywords:** domestication, natural selection, RMR, *Salmo salar*, semi‐natural, SMR

## Abstract

Escaped farmed Atlantic salmon interbreed with wild Atlantic salmon, leaving offspring that often have lower success in nature than pure wild salmon. On top of this, presence of farmed salmon descendants can impair production of wild‐type recruits. We hypothesize that both these effects connect with farmed salmon having acquired higher standard metabolic rates (SMR, the energetic cost of self‐maintenance) during domestication. Fitness‐related advantages of phenotypic traits associated with both high SMR and farmed salmon (e.g., social dominance) depend on environmental conditions, such as food availability. We hypothesize that farmed offspring have an advantage at high food availability due to, for example, dominance behavior but suffer increased risks of starvation when food is scarce because this behavior is energy‐demanding. To test these hypotheses, we first compare embryo SMR of pure farmed, farmed‐wild hybrids and pure wild offspring. Next, we test early‐life performance (in terms of survival and growth) of hybrids relative to that of their wild half‐siblings, as well as their competitive abilities, in semi‐natural conditions of high and low food availability. Finally, we test how SMR affects early‐life performance at high and low food availability. We find inconclusive support for the hypothesis that domestication has induced increased SMR. Further, wild and hybrid juveniles had similar survival and growth in the semi‐natural streams. Yet, the presence of hybrids led to decreased survival of their wild half‐siblings. Contrary to our hypothesis about context‐dependency, these effects were not modified by food availability. However, wild juveniles with high SMR had decreased survival when food was scarce, but there was no such effect at high food availability. This study provides further proof that farmed salmon introgression may compromise the viability of wild salmon populations. We cannot, however, conclude that this is connected to alterations in the metabolic phenotype of farmed salmon.

## 1 INTRODUCTION

Feral domestic animals commonly constitute a threat to the viability of wild populations since their hybridization with wild individuals can disrupt local adaptations and reduce population fitness (Frankham, 2008; Laikre, Schwartz, Waples, & Ryman, 2010). Currently, there is concern for wild Atlantic salmon (*Salmo salar*) populations because of the expansion of the Atlantic salmon aquaculture industry and the accompanying increase in number of escapees breeding in nature (Forseth et al., 2017; Glover et al., 2017; Wringe et al., 2018). Wild salmon populations have evolved adaptations that are beneficial in their local environments (Fraser, Weir, Bernatchez, Hansen, & Taylor, 2011; Garcia de Leaniz et al., 2007; O'Toole et al., 2015). Hence, when interbreeding with escaped farmed salmon causes genetic introgression into wild populations (Glover et al., 2013; Karlsson, Diserud, Fiske, & Hindar, 2016; Skaala, Wennevik, & Glover, 2006), this increases the number of mal‐adapted individuals in nature (Bolstad et al., 2017). Farmed salmon and their descendants often have lower success in nature than wild salmon (Fleming et al., 2000; McGinnity et al., 2003), and genetic introgression of farmed salmon may therefore lead to a temporal or permanent decline in fitness of the wild populations affected. On top of this, the presence of individuals with farmed salmon ancestry can entail decreased production of seaward migrants of the wild type (Fleming et al., 2000).

Both the reduced success of farmed salmon and their descendants in nature, and the negative effect of their presence for production of wild salmon, are likely related to genomic and phenotypic alterations that have occurred during the domestication process (Bolstad et al., 2017; Liu et al., 2017). Among the phenotypic alterations reported are increased growth rates (Gjedrem, 2000; Harvey et al., 2016; Solberg, Skaala, Nilsen, & Glover, 2013a) and changes in behavior, such as decreased response to predators, and increased aggression and social dominance (Einum & Fleming, 1997; Houde, Fraser, & Hutchings, 2010a; Johnsson, Höjesjö, & Fleming, 2001). Decreased anti‐predator response poses an obvious disadvantage in nature and could contribute to the reduced success of farmed salmon and their descendants. On the other hand, rapid growth and aggressive and dominant behavior could give farmed salmon a competitive advantage that enable them to displace wild salmon from territories with, for example, good feeding opportunities and may explain the finding of reduced production of wild salmon (Fleming et al., 2000).

Standard metabolic rate (SMR, defined as the energetic cost of self‐maintenance, reviewed in Burton, Killen, Armstrong, & Metcalfe, 2011; Metcalfe, Leeuwen, & Killen, 2016) is another trait that may have been affected by the domestication process. Conditions characteristic of the farm environment, including high, predictable food availability and structurally simple habitats, concur with conditions reported to be advantageous for individuals with high SMR (Bozinovic & Sabat, 2010; Derting, 1989; Reid, Armstrong, & Metcalfe, 2012). Conversely, organisms in habitats with low food availability and/or predictability, conditions commonly found in nature, are hypothesized to evolve low SMR (reviewed in Chown & Gaston, 1999; Cruz‐Neto & Bozinovic, 2004). Additionally, SMR is connected with a suite of phenotypes similar to some that are typical for farmed salmon, such as rapid growth rate, dominance and risk‐prone behavior (Killen, Marras, Ryan, Domenici, & McKenzie, 2012; Metcalfe, Taylor, & Thorpe, 1995; Millidine, Metcalfe, & Armstrong, 2009). We therefore hypothesize that farmed Atlantic salmon have increased SMR compared to wild salmon. We test this by measuring SMR of embryos (eyed eggs) resulting from experimental crosses of farmed and wild salmon.

The advantages of phenotypic traits commonly associated with both farmed salmon and high SMR rely heavily on environmental conditions. For instance, farmed or growth hormone implanted salmon rapidly outgrow salmon of the wild type in captivity with high and reliable access to food (Solberg, Zwei, Nilsen, & Glover, 2013b; Sundt‐Hansen et al., 2012). This trend is much less prominent and may even be reversed under natural conditions where food access is typically less reliable and scarce (Glover, Solberg, Besnier, & Skaala, 2018; Reed et al., 2015; Sundt‐Hansen et al., 2012). Hence, we hypothesize that the performance of farmed salmon offspring, as well as their ability to outcompete wild salmon, depends on food availability. Specifically, we expect that farmed offspring have an advantage in conditions with high food availability due to their high growth potential and aggressive and dominant behavior. Conversely, we expect that farmed offspring have a disadvantage at low food availability when they cannot realize their growth potential and may suffer increased risks of starvation because of their energy‐demanding behavior. How food regime affects benefits versus costs of having high SMR differs among published studies. When food is readily available, high SMR has been reported either to be advantageous or to have no performance effect. In contrast, under conditions of food shortage high SMR has been found to be advantageous, disadvantageous, or to have no performance effect (Auer et al., 2018; Bochdansky, Grønkjær, Herra, & Leggett, 2005; Zeng et al., 2017).

In this study, we test how food availability affects the success of offspring of farmed and wild salmon, as well as their competitive ability, in an experiment with farmed‐wild hybrids and pure wild salmon in 40 semi‐natural streams. We manipulate food availability (high versus low) and competitive regime (allopatry: wild and hybrid juveniles alone versus in sympatry: wild and hybrid juveniles together) during the critical period for survival following juvenile emergence from the gravel and onset of exogenous feeding (Einum & Fleming, 2000; Einum, Sundt‐Hansen, & Nislow, 2006; Elliott, 1984). We compare the performance of juveniles (in terms of survival and growth) across the food availability and competition treatments. In doing so, we also test whether the previously found negative impact on early survival of wild offspring inflicted by the presence of pure farmed salmon offspring (Sundt‐Hansen, Huisman, Skoglund, & Hindar, 2015) extends to hybrids. Finally, we test for effects of different levels of SMR on juvenile performance at high and low food availability by collating data on family‐level embryo SMR with data on performance at the individual and family level.

## 2 MATERIALS & METHODS

### 2.1 Experimental crosses

#### 2.1.1 Parent fish

Atlantic salmon from two Norwegian populations were used as representatives for wild salmon. We sampled gametes from 20 adults (10 of each sex, Supporting Information Table S1) from the River Surna (Central Norway, 63.06°N, 9.14°E) that were caught during broodstock collection in autumn 2012. Gametes from the River Imsa (Southwestern Norway, 58.91°N, 5.95°E) were taken from 22 adults (11 of each sex, Supporting Information Table S1) caught during 2013 in a fish trap when they returned to the river to spawn.

Atlantic salmon from the Norwegian breeding company AquaGen were used as our farmed salmon representative. This population was originally founded from 41 Norwegian wild populations in 1971–1974 and had been subject to domestication and artificial selection for 10–11 generations when we made our crosses in 2012 and 2013. The River Surna was one of the founding populations of the AquaGen breeding strains (Gjøen & Bentsen, 1997). The AquaGen material used to make crosses in 2012 originated from gametes stripped from 20 farmed adults (10 of each sex, Supporting Information Table S1), whereas the material used to make crosses in 2013 originated from 22 farmed adults (11 of each sex, Supporting Information Table S1).

#### 2.1.2 Crosses

Following stripping, gametes were kept on ice in plastic containers enriched with oxygen for shipment and storage until all crosses were done at the NINA Research Station Ims (hereafter referred to as “Ims”). All fertilizations were performed within the same day (8 November 2012 and 29 November 2013). Eggs from each wild and farmed female were fertilized with sperm from one wild and one farmed male so that each pure wild (ww) and pure farmed (ff) family were half‐siblings with two hybrid families; one with a farmed mother and a wild father (fw), and one with a wild mother and a farmed father (wf). The crosses are hereafter called “types”. The crosses performed using gametes from Surna and AquaGen in 2012 gave 40 full‐sibling family groups. This part of the fish material will be referred to as “Surna‐AquaGen”. The crosses made with AquaGen and Imsa gametes in 2013 resulted in 44 full‐sibling family groups, which are hereafter referred to as “Imsa‐AquaGen”.

Eggs and juveniles originating from these crosses were used to conduct two different sets of experiments: O_2_ consumption was measured at the embryo (eyed egg) stage using both the Surna‐AquaGen and the Imsa‐AquaGen material, whereas tests of juvenile performance under semi‐natural conditions were undertaken only with the Imsa‐AquaGen material.

Due to low fertilization success or high mortality at the egg or juvenile (alevin) stage, some families are not represented in the experiments that constitute this study. See the descriptions of experiments below for information on numbers of families included in the different parts of the study.

### 2.2 Measurement of embryo standard metabolic rate

Eyed embryos used in the SMR measurements were shipped from Ims to the Norwegian University of Science and Technology (NTNU) on 5 February 2013 and 24 February 2014. At NTNU, the families were kept in separate petri dishes at 5°C. Representatives from all families had their SMR measured as the rate of O_2_ consumption in a closed system, the Surna‐AquaGen material during 7–10 February 2013 and the Imsa‐Aquagen material during 26 February to 3 March 2014. Total O_2_ consumption in each sample was measured with a micro‐cathode oxygen electrode (model 1320) connected to an oxygen meter (model 781; Strathkelvin Instruments Ltd, Glasgow, UK).

O_2_ consumption in the Surna‐AquaGen and the Imsa‐AquaGen materials was measured with similar procedures. Individual embryos from the Surna‐AquaGen material were placed inside 2 ml syringes containing 10°C oxygenated water. The water had been transported from Ims and was filtered with Sterivex 0.2‐µm filters. Syringes were sealed with warm wax and kept for 2.2–4 hr at 10°C. Sixteen syringes were not loaded with embryos, and these functioned as controls to account for microbial metabolism. For the Imsa‐AquaGen material, five embryos per family were placed inside separate 13 ml glass vials that contained aerated 10°C synthetically prepared water (COMBO water, Kilham, Kreeger, Lynn, Goulden, & Herrera, 1998) and sealed with plastic caps under water and then with warm wax. Sixteen glass vials were used as controls. Vials were placed on a rotation table to prevent formation of O_2_ gradients and were kept at 10°C for 2–2.5 hr. For both the Surna‐AquaGen and the Imsa‐AquaGen material, the O_2_ content was measured by inserting water into the chamber of the oxygen electrode using a syringe and recording values once readings had been stable for 5 s. Measurements were made both from a sample of the water from which the vials or syringes were filled at the time when the embryos were loaded into the syringes or glass vials, and again from the vials or syringes containing the eggs at the end of the measurement period.

Oxygen consumption was successfully measured on 6–9 individual embryos from each of 5 pure Surna families, 8 hybrid families with AquaGen mother and Surna father, 7 hybrid families with Surna mother and AquaGen father and 10 pure AquaGen families. The O_2_ consumption of 11 pure Imsa families, 11 of the hybrid families with AquaGen mother and Imsa father, 10 of the hybrid families with Imsa mother and AquaGen father and 9 pure AquaGen families was measured in 3–5 replicates per family.

All eggs were weighed to the nearest 0.1 mg at the end of each day of O_2_ consumption measurements for the Surna‐AquaGen material and on the day following O_2_ consumption measurements for the Imsa‐AquaGen material. These measurements constitute the basis for the mean embryo masses reported in Supporting Information Table S1. A subsample of embryos was also photographed for later linear measurements to calculate the relationship between embryo mass and volume.

Total O_2_ consumption (mg) of the embryos was calculated from the measured decline in O_2_ concentration and the water volume (i.e., excluding the volume displaced by eggs) in the containers. Control measurements were used to correct for microbial metabolism. The results showed a decline in estimated O_2_ consumption h^−1^ with increasing duration of the measurement period, likely caused by diffusion of O_2_ into the containers. We could therefore not use O_2_ consumption h^−1^ as a direct measure of SMR. To account for differences in duration of the measurement period among samples, and variation in embryo size, we estimated mass‐specific SMR as residuals from linear models where total O_2_ consumption (mg) was regressed against the duration of the measurement period and egg mass (Surna‐AquaGen: *r* ^2^ = 0.36, *F*_2, 291 =_83.31, *p* < 0.001; Imsa‐AquaGen: *r* ^2^ = 0.31, *F*_2, 184_ = 41.64, *p* < 0.001). Oxygen consumption and egg mass were log_10_‐transformed prior to these regressions to linearize the relationship between them (Supporting Information Figure S1).

### 2.3 Survival and growth of juveniles in a semi‐natural environment

To examine survival effects of competition between wild offspring (Imsa × Imsa) and both types of hybrids (Imsa × AquaGen, either with farmed or wild mother) at different levels of food availability, we set up an experiment in 40 semi‐natural stream channels. The stream channels were 4.5 m long, 24 cm wide, had a water level of 10–15 cm, and gravel substratum suitable for salmon juveniles (see e.g., Sundt‐Hansen et al., 2015). Each channel had a mesh at both ends providing a confined environment. Food availability was manipulated by keeping 20 of the channels dry for five weeks immediately prior to initiation of the experiment, whereas water was allowed to run through the other channels during the same period to allow benthos establishment (cf. Einum & Fleming, 1999). To further ensure a contrast in food availability between the low and high food treatment, chironomid larvae were provided at 50% of the maintenance diet (the energy sufficient to maintain a juvenile without any change in its energy content) for the low food treatment and at 100% of the maximum diet in the high food treatment (calculated following Elliott, 1976). Chironomid larvae were introduced as semi‐defrosted blocks of approximately 0.3 cm^3^ (1 block per day per stream channel for the low food treatment vs. 3 for the high food treatment) that were spread by the water current so that food was available throughout the full length of each stream channel. Pre‐trial tests confirmed that this procedure ensured a consistent distribution of food across the stream channels. Salmon juveniles from six families of the wild type as well as six from each of the two hybrid types were used (Supporting Information Table S2). A total of 36 individual juveniles were stocked in each stream channel on 5 May 2014 (Supporting Information Table S2), a few days before predicted median timing of emergence from the gravel (Crisp, 1981, 1988). Surviving fish were recaptured using dip nets on 10 June 2014 and assigned to their respective families using SNP analyses (see description below). The sampling was continued until depletion to ensure recapture of all fish.

Upon experiment termination, holes were found in the mesh of eight stream channels, all from the low food treatment. These replicates were excluded. The number of replicates omitted at the low food treatment was as follows: one for each of the hybrid types in allopatry, two for wild fish in allopatry, two for the sympatric treatment with wf hybrids and two for the sympatric treatment with fw hybrids (Supporting Information Table S2). In six additional replicates, one individual had been able to move to the neighboring stream channel (identified by genetic analyses). These six individuals were excluded from the analysis but the replicates were retained as the loss or gain of one individual would have limited effect on the remaining individuals.

#### 2.3.1 Genotyping and parental assignment

We extracted total genomic DNA from the 24 broodfish used to make the Imsa‐AquaGen material, as well as from 942 surviving juveniles using the DNeasy kit from Qiagen (Hombrechtikon, Switzerland). Ninety‐six SNPs (Bourret et al., 2013) were genotyped with an EP1™ 96.96 Dynamic array IFCs (Fluidigm, San Francisco, CA, USA). Fifteen of these SNPs were located in the mitochondrial genome (Karlsson, Moen, & Hindar, 2010).

All broodfish were successfully genotyped at the 81 nuclear SNP loci and the 15 SNPs in the mitochondrial DNA. Nine hundred and twenty‐one offspring were successfully genotyped at more than 95% of the 81 nuclear SNPs, and 16 were genotyped for 59–76 nuclear SNPs. Five individuals had poor genotyping and were excluded from further analyses. Of the remaining 937 offspring assigned to parents, all but four were genotyped at all of the 15 mtDNA SNPs.

Parental assignment was conducted by a genotype exclusion approach allowing for mismatches (Vandeputte, Mauger, & Dupont‐Nivet, 2006) and crosses between broodfish regardless of sex and registered crossings (Karlsson, Saillant, Bumguardner, Vega, & Gold, 2008). The latter was done as a check of the assignment power. Because mitochondrial DNA (mtDNA) is maternally inherited, we checked for possible assignment errors by comparing the haplotype of the offspring with that of the assigned mother using the 15 mitochondrial SNPs.

All 937 offspring were unambiguously assigned to a parental pair when allowing for all possible crossings between broodfish regardless of sex, and the assigned parental pairs were in agreement with the actual crossings. We identified five different haplotypes in the mtDNA, and females and their assigned offspring had the same haplotypes.

### 2.4 Statistics

All statistics were conducted in R v.3.5.1. (R Core Team, 2018). Fixed effects in linear mixed effects models (LMM) and in generalized linear mixed effects models (GLMM), both from the lme4 package (Bates, Mächler, Bolker, & Walker, 2015), were assessed using a backwards selection procedure (Zuur, Ieno, Walker, Saveliev, & Smith, 2009). Starting with a full model fitted with maximum likelihood (ML), fixed factors were sequentially removed, and the resulting simpler models were compared with the preceding models using Akaike's information criterion (AIC). The removal of non‐significant terms was done consecutively until the removal of further terms resulted in an increase in AIC ≥ 2.

#### 2.4.1 Standard metabolic rate

Differences in mass‐specific standard metabolic rate (SMR) of embryos resulting from different crosses between farmed and wild adults (farmed × farmed, wild × wild, and reciprocal hybrids) at the eyed embryo stage were tested using LMMs. Different LMMs were fitted for the Surna‐AquaGen and the Imsa‐AquaGen data. Both models included the main fixed effects of female type (*FT*) and male type (*MT*) and their interaction. Since each female and male was represented by several offspring (full and/or half‐siblings), we added female identification (femID) and male identification (maleID) as random intercepts. Thus, the initial LMM models for embryo SMR can be represented as:

$$\text{SMR}=\alpha +\beta_{1}FT+\beta_{2}MT+\beta_{3}FTMT+b_{\text{femID}}+b_{\text{maleID}}+\varepsilon ,$$

where *α* is the intercept, *β* are fixed factors, b are random factors and ε is a random error.

#### 2.4.2 Juvenile performance in a semi‐natural environment—effects of competition, food availability and standard metabolic rate

Juvenile survival was modeled in two different ways. First, we tested whether there were differences in survival *among* the three types (ww, fw, and wf) and if any such effect depended on food availability (high and low), *within* each of the two competition treatments (allopatry and sympatry). At the same time, we tested if the mean family mass‐specific SMR (family‐level SMR) or mean family embryo mass had an effect on survival, and whether any such effect depended on food availability. This was done by modeling the survival (*S*) of families from the different types in allopatry and in sympatry in two separate binomial GLMMs that included the main effects of type (*T*), family‐level SMR (*fSMR*), family‐level embryo mass (*fEM*, mean centered), food availability treatment (*F*), and the interaction between *F* and the other main effects. Family (fam) and stream channel (ch) were included as random intercepts to take into account possible block effects and that each family was represented in several replicates. Thus, the structure of the starting GLMMs was as follows:

$$\text{logit}\left(S\right)=\alpha +\beta_{1}T+\beta_{2}fSMR+\beta_{3}fEM+\beta_{4}F+\beta_{5}TF+\beta_{6}FfSMR+\beta_{7}FfEM+b_{\text{fam}}+b_{\text{ch}}+\varepsilon$$

Secondly, we tested if the competition (ww, wf and fw in allopatry, ww and wf in sympatry, ww and fw in sympatry) , food treatments, family‐level SMR or any of the interactions between these main effects had an effect on survival *within* each type. This was done by modeling the effect of competition and food treatment on survival (*S*) of wild offspring and each of the two types of hybrids in three separate binomial GLMMs, one for each type. All initial models included the main effect of the food availability treatment (*F*), competition (C), family‐level SMR (fSMR) and the interaction between these, as well as the main effect of family‐level embryo mass (fEM, mean centered) and its interaction with F and C. Random factors for family (fam) and stream channel (ch) were also included. The structure of these models was:

$$\begin{array}{cc} \text{logit}\left(S\right)= & \alpha +\beta_{1}F+\beta_{2}C+\beta_{3}fSMR+\beta_{4}fEM+\beta_{5}FC+\beta_{6}FfSMR+\beta_{7}CfSMR\\ \;+\beta_{8}FCfSMR+\beta_{9}FfEM+\beta_{10}CfEM+\beta_{11}FCfEM\;+b_{\text{fam}}+b_{\text{ch}}+\;\varepsilon . \end{array}$$

Variation in final mass among juveniles of different types was modeled in a LMM where final individual mass (*FM*, ln‐transformed) was the response variable. Family‐level embryo mass (*fEM*, ln‐transformed) was included as a co‐variate to take into account variation in start weight of juveniles from different families. Also included were the main effects of type (T), food availability treatment (F), competition (C: sympatry or allopatry), the final number of surviving juveniles (N) in each stream channel and family‐level SMR (fSMR), as well as the interactions between T and F and T and C, as well as their interactions with fSMR. Similar to the GLMM models, stream channel (ch) and family (fam) were included as random factors. Thus, the initial LMM model can be represented as:

$\begin{array}{cc} \ln\left(\mathrm{FM}\right) & =\alpha +\beta_{1}\ln\left(\mathrm{fEM}\right)+\beta_{2}N+\beta_{3}T+\beta_{4}F+\beta_{5}C+\beta_{6}fSMR+\beta_{7}TF\\ & +\beta_{8}TC+\beta_{9}fSMRT+\beta_{10}fSMRC+\beta_{11}fSMRF+\beta_{12}fSMRTC\\ & +\beta_{13}fSMRFC+\beta_{14}fSMRTF+b_{\mathrm{fam}}+b_{\mathrm{ch}}+\varepsilon \end{array}$

## 3 RESULTS

### 3.1 Standard metabolic rate

**Figure 1:**
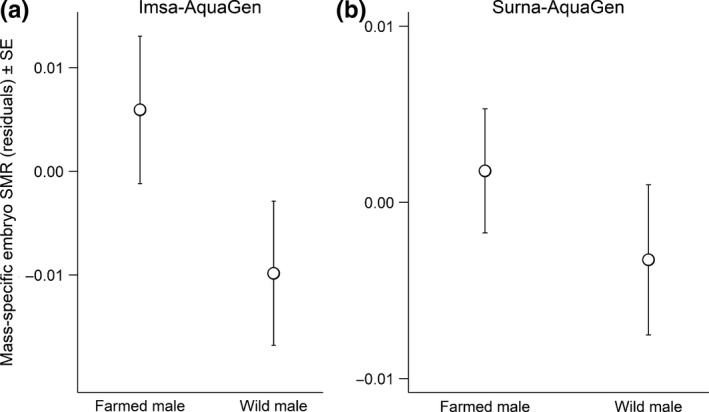
Estimated residual mean SMR ± *SE* of Atlantic salmon embryos of farmed and wild males resulting from crosses between the farmed AquaGen strain and (a) the river Imsa population and (b) the river Surna population. Embryos of farmed and wild females are merged because their SMRs did not differ according to model selection. In the Imsa‐AquaGen crosses (a) embryos of farmed males had higher SMRs than those of wild males (*p* = 0.012). According to the model selection, there were no significant differences in the Surna‐AquaGen crosses (b)

The main effect of male type was retained in the model that best described variation in mass‐specific SMR in embryos from the Imsa‐AquaGen crosses, whereas female type and the interaction between female and male type could be excluded. Thus, mass‐specific SMR did not depend significantly on whether an egg was produced by a farmed or wild female. However, it was lower in embryos fertilized with sperm from a wild male than from a farmed male (difference in intercept estimate = −0.016, *SE* = 0.006, *t* = −2.54, *p* = 0.012; Figure 1a).

In the Surna‐AquaGen crosses, none of the main effects or interactions were retained in the model that best explained variation in the mass‐specific SMR of embryos. Thus, mass‐specific SMR of the Surna‐AquaGen embryos was not significantly influenced by whether the parents were of farmed or wild origin (Figure 1b).

### 3.2 Juvenile performance in a semi‐natural environment

#### 3.2.1 Differences in survival among wild and hybrid crosses within competition treatments

According to the model selection, only the main effect of the food availability treatment influenced survival in allopatry. Thus, the survival of wild and hybrid juveniles did not differ significantly in allopatry but was significantly lower in the low food treatment (64%) than in the high food treatment (87.5%) (difference in parameter estimates given on the logit scale = −1.37, *SE* = 0.48, *Z* = −2.85, *p* = 0.004). Furthermore, family‐level SMR and embryo mass had no significant effect on survival across.

The corresponding best model for survival in sympatry included only the main effect of family embryo mass, with a positive effect of large mass (slope estimate given on the logit scale: 18.06, *SE* = 7.52, *Z* = 2.41, *p* = 0.02). There was no significant difference in survival between the food availability treatments or among the types, and there was no effect of family‐level SMR.

#### 3.2.2 Differences in survival within wild and hybrid crosses among competition treatments

**Figure 2:**
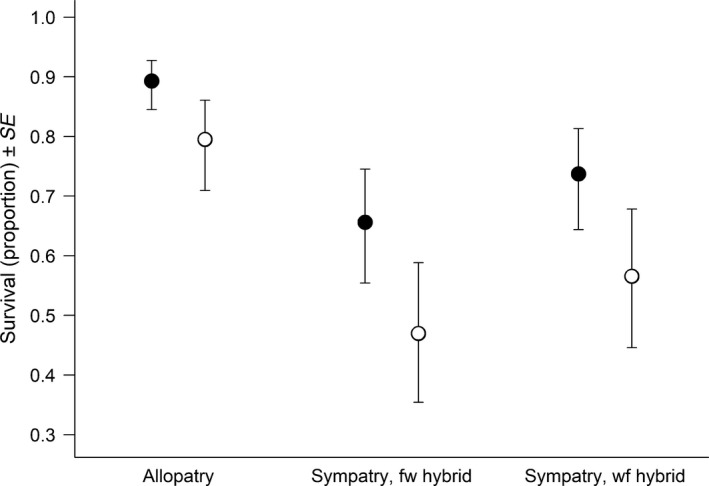
Survival of wild Atlantic salmon juveniles of the Imsa population in semi‐natural streams at high (closed) and low (open) food availability when in allopatry and sympatry with hybrids with farmed mother and wild father (fw) or hybrids with wild mother and farmed father (wf). The values are back‐transformed estimates from the binomial GLMM that was best according to model selection

**Figure 3:**
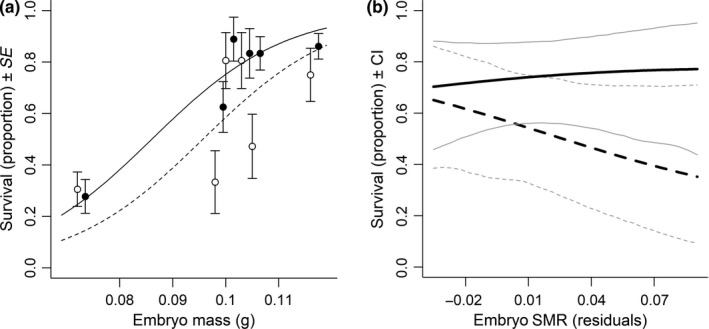
Relationships for the wild salmon juveniles of the Imsa population between (a) survival and family‐level embryo mass at high (solid, filled) and low (dotted, open) food availability plotted together with mean ± *SE* values for each family, and (b) survival ± CI (gray) and family‐level embryo SMR at high (solid) and low (dotted) food availability after correcting for survival effects of family‐level embryo mass. Estimated survival effects of embryo mass are centered and plotted on mean values of the raw data. All relationships are back‐transformed estimates from the best GLMM for the treatment where wild salmon were in sympatry with hybrids with farmed father. The pattern was consistent across treatments (wild salmon in allopatry and in sympatry with both types of hybrids, shown in Table 1)

For the survival of wild juveniles, the best model included the main effects of the food availability treatment, competition, family‐level embryo mass, family‐level SMR, and the interaction between food availability and family‐level SMR. According to this model, wild offspring had significantly lower survival when they competed with either of the hybrid types (Table 1, Figure 2) than when they were in allopatry. Survival of wild juveniles increased significantly with increasing mean family embryo mass (Table 1, Figure 3a). Also, the relationship between family‐level SMR and survival differed among the food treatments for the wild families (Table 1, Figure 3b). Specifically, families with high SMR had lower survival than families with low SMR when food was limited. When food was abundant, SMR had no significant effect on the survival of individuals from the wild families.

**Table 1:** Parameter estimates from three statistical models that best describe survival of Atlantic salmon juveniles with wild parents (*n* = 108, no. stream channels = 18), farmed mother and wild father (*n* = 78, no. of stream channels = 13) and wild mother and farmed father (*n* = 78, no. of stream channels = 13) in allopatry and sympatry and at high and low food availability in semi‐natural channels. For juveniles with two wild parents, the estimated slopes for survival effects of family‐level embryo SMR at high and low food availability treatments and for family‐level embryo mass (mean centered) are also given. All values are on logit scale and given as treatment contrasts

	Estimate ± *SE*	*Z*	*p*‐value
**Wild (ww)**			
Intercept (allopatry, high food)	2.12 ± 0.43	4.97	˂0.001
Sympatry, fw	−1.48 ± 0.45	−3.28	0.001
Sympatry, wf	−1.09 ± 0.45	−2.40	0.016
Low food	−0.77 ± 0.39	−1.97	0.049
Family SMR (high food)	3.51 ± 8.34	0.42	0.67
Family SMR:food (low food)	−14.52 ± 6.55	−2.22	0.03
Family embryo mass	79.57 ± 22.67	3.51	<0.001
**Hybrid with farmed mother (fw)**			
Intercept (high food)	1.78 ± 0.41	4.31	<0.001
Low food	−1.35 ± 0.58	−2.33	0.02
**Hybrid with wild mother (wf)**			
Intercept (high food)	1.65 ± 0.52	3.18	0.002
Low food	−1.63 ± 0.80	−2.03	0.04

For both types of hybrids (both those with farmed mother and wild father, and those with wild mother and farmed father), the corresponding best models included only the main effect of food availability. According to these models, survival was significantly lower at the low food treatment (60.7%; 50.5%) than at the high (85.6%; 84%) for wf and fw families, respectively (Table 1). Thus, the survival of the hybrids was not significantly influenced by the presence of wild offspring and did not depend on the family‐level SMR or embryo mass.

#### 3.2.3 Growth

**Figure 4:**
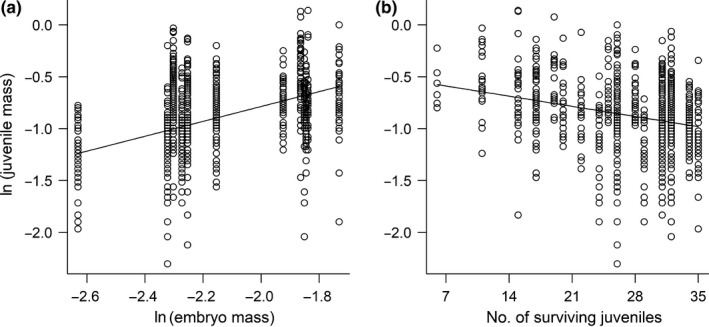
Estimated relationship between individual mass (g, ln‐transformed) of Atlantic salmon juveniles in semi‐natural stream channels at the termination of the experiment and (a) family‐level embryo mass (g, ln‐transformed), and (b) number of surviving juveniles in each stream channel plotted on raw data

According to model selection, only the main effects of family embryo mass (ln‐transformed) and the final number of surviving fish in each stream channel had an effect on the individual final mass in the stream channel. Specifically, there was a positive relationship between family embryo mass and final mass and a negative relationship between the final number of survivors in each stream channel and final mass (Table 2, Figure 4). Hence, the food availability treatments were less important for growth than both family‐level embryo mass and the final number of fish left in each stream channel. Furthermore, there was no significant effect of the competition treatment, family‐level SMR and no significant differences in final mass among the different types after correcting for variation in embryo mass.

**Table 2:** Summary of the statistical model that best describe the relationship between mass (g, ln‐transformed) of Atlantic salmon juveniles in semi‐natural stream channels (*n* = 799, no. of stream channels = 32, no. of families = 18) at the end of the experiment and mean family embryo mass (g, ln‐transformed), and the number of juveniles left in each stream channel upon experiment termination

	Estimate ± *SE*	*df*	*T*	*p*‐value
Intercept	1.03 ± 0.36	22.5	2.86	0.009
Family embryo mass	0.72 ± 0.16	19.4	4.5	<0.001
No. of juveniles	−0.01 ± 0.004	33.1	−3.80	<0.001

## 4 DISCUSSION

This study advances the concern that feral domestic animals impact wild populations negatively since we found that the presence of farmed‐wild Atlantic salmon hybrids led to decreased early survival of their wild half‐siblings under controlled, semi‐natural conditions. This negative influence of hybrid offspring on survival of wild juveniles was, at least partly, caused by genes associated with farmed salmon since it prevailed irrespective of whether the wild juveniles competed with half‐siblings from wild or farmed mothers. That is, irrespective of maternal effects due to, for example, egg size differences between farmed and wild mothers. Combined with results from earlier studies (Fleming et al., 2000; Sundt‐Hansen et al., 2015), this demonstrates that genetic introgression of farmed salmon may represent a direct cost to wild populations by imposing increased mortality on genetically wild individuals at the critical early life stage.

As expected, the survival of both wild and hybrid fry was lower at low food availability than at high food availability. Contrary to our predictions, food availability did not influence the effect that hybrids had on the survival of wild fry, thereby indicating that hybrids were as strong competitors at low food availability as they were at high. Furthermore, the relative survival of wild and hybrid fry in sympatry and in allopatry did not differ between the food availability treatments. Thus, despite the farmed salmon strain used in this experiment having adapted to high food availability for 11 generations, their hybrid offspring were able to perform as well as, and even outcompete wild salmon, under low food availability.

The impact of SMR on survival among wild families depended on food availability. At high food availability, family‐level SMR had no effect on survival, while at low food availability there was a negative relationship between family‐level SMR and survival. Thus, in accordance with findings in Bochdansky et al. (2005), there was a cost of having high SMR when food availability was low, which could potentially have been caused by high energetic needs necessary to maintain a high SMR (Millidine et al., 2009). We did not find any effect of family‐level SMR on survival of hybrid families, but this could be a result of the narrow range in residual SMR among the hybrid families that were stocked in the stream channels (fw: −0.071 to 0.007, wf: −0.025 to 0.039) compared to that of the wild families (ww: −0.035 to 0.091). Moreover, recent findings suggest that other traits of the metabolic phenotype, such as maximum metabolic rate (MMR) and aerobic scope (AS, the difference between SMR and MMR), could be more important predictors for both growth and survival under contrasting environments than SMR (Auer et al., 2018; Auer, Salin, Rudolf, Anderson, & Metcalfe, 2015, 2016; Závorka et al., 2017). Thus, future studies of metabolic rates in farmed versus wild salmon should be extended to include other traits of the metabolic phenotype.

Under conditions with high competition, the large juveniles that hatch from large eggs often have a competitive advantage (Hutchings, 1991; Robertsen, Skoglund, & Einum, 2013). Thus, to make sure that variation in egg sizes did not underlie our results, we tested and controlled for effects of egg size in all our statistical models. As expected based on the relatively high fish densities in the stream channels, a general positive relationship between egg mass and survival prevailed across food availability and competition treatments among our wild families. For the hybrid families, however, there was no significant effect of egg size on survival.

In contrast to several published results showing that hybridization between farmed and wild Atlantic salmon results in offspring that display lower survival in nature than wild offspring (McGinnity et al., 2003; Reed et al., 2015; Skaala et al., 2012), we did not detect significantly lower survival of the hybrid juveniles than that of their wild half‐siblings. This was true both when they were alone (allopatry) and in competition (sympatry) with their wild half‐siblings under near‐natural conditions. This finding is consistent with that of Sundt‐Hansen et al. (2015) where fry with two farmed parents even had higher survival than fry with wild parents under conditions similar to those in our experiment. One possible explanation for the discrepancies between the results from these two studies and other studies is that the other studies have generally dealt with later life stages. It is therefore possible that the performance of farmed offspring in nature at the early juvenile stage examined in this study may not be any poorer than that of their wild counterparts and that the farmed offspring thus fail at a later stage. Another plausible explanation for the lack of difference in hybrid relative to wild offspring survival in the present study is that the semi‐natural conditions did not fully replicate nature. For example, there were no predators present. Studies reporting lower anti‐predatory response of farmed compared to wild offspring (Einum & Fleming, 1997; Houde, Fraser, & Hutchings, 2010b) suggest that they could be more vulnerable to predation than their wild counterparts. Thus, if there had been predators present in our study we may have seen lower survival of the hybrids relative to that of the wild individuals.

We found no difference in growth among the farmed‐wild hybrids and the wild juveniles. It appears that hybrid offspring were not able to utilize the higher growth potential from their farmed ancestry (Gjedrem, 2000) under the conditions of this experiment, similar to that seen for growth hormone implanted Atlantic salmon in nature (Sundt‐Hansen et al., 2012). There was also no difference in growth rate between our high and low food treatment, which is likely ascribed to higher mortality in the low food treatment. The final number of individuals left in each stream channel had a significant negative effect on body mass. Thus, the per capita food availability could have ended up similar in the two treatments. The lack of difference in growth across food treatments suggests that the increased mortality in the low food versus high food treatment probably manifested itself early during the course of the experiment (cf. Einum et al., 2006).

Our laboratory tests of the hypothesis that farmed Atlantic salmon have acquired increased mean levels of SMR compared to that of wild salmon gave inconclusive results. Embryos of farmed males had significantly higher SMR than embryos of wild males in the crosses between farmed salmon and wild salmon of the Imsa population, but there was not a similar finding in the Surna population. Moreover, SMR did not differ significantly between embryos of farmed females and females of the wild populations. This is, however, in line with the previously reported finding that phenotypic effects of farmed introgression vary among wild Atlantic salmon populations (Bolstad et al., 2017).

We emphasize that the methodology we employed to measure SMR is coarse. Yet, it should provide conservative results. For example, since the containers used to measure SMR were not totally impermeable to O_2_, a decline in the O_2_ concentration due to embryo metabolism would continuously be counteracted by O_2_ diffusing in, leading to an overall underestimation of the O_2_ consumption. This tendency would be more pronounced in containers containing embryos with high SMR since the O_2_ concentration in these would decrease at a faster rate than in containers containing embryos with low SMR. Thus, if a more precise methodology had been employed, larger differences among the Atlantic salmon types in this study could possibly have been detected.

Our results show that descendants of domesticated organisms can induce increased mortality of genetically wild individuals in early life. The resulting decrease in production of recruits may obviously impact the viability of wild populations negatively. In addition, presence of domesticated descendants and an accompanying increase in mortality of wild‐type juveniles could affect the adaptive landscape, potentially resulting in unforeseen changes to the wild genotype.

## CONFLICT OF INTEREST

None declared.

## AUTHORS CONTRIBUTION

GR, DR, SE, IAF, OU, EK, and KH contributed significantly to the conception and design of the work. GR, DR, TA, and LSH performed different parts of the experiments. SK planned and led the work with parentage assignment. GR, SE, and IAF contributed significantly to the analyses and interpretation of the data. GR, SE, and IAF drafted the manuscript. All authors revised the intellectual contents of the manuscript critically and agree to be accountable for all aspects of this work.

## DATA ACCESSIBILITY

Data available from the Dryad Digital Repository: https://doi.org/10.5061/dryad.17bq5d8
