# Corrigendum

**DOI:** 10.1002/ece3.5974

**Published:** 2020-01-29

In “Can variation in standard metabolic rate explain context‐dependent performance of farmed Atlantic salmon offspring?” which was published in issue 1, January 2019, the authors have requested to add an additional footnote to Table 1.

**Table 1:** Parameter estimates from three statistical models that best describe survival of Atlantic salmon juveniles with wild parents (n = 108, no. stream channels = 18), farmed mother and wild father (n = 78, no. of stream channels = 13) and wild mother and farmed father (n = 78, no. of stream channels = 13) in allopatry and sympatry and at high and low food availability in semi‐natural channels. For juveniles with two wild parents, the estimated slopes for survival effects of family‐level embryo SMR at high and low food availability treatments and for family‐level embryo mass (mean centered) are also given. All values are on logit scale and given as treatment contrasts

	Estimate ± *SE*	*Z*	*p*
Wild (ww)			
Intercept (allopatry, high food)	2.12± 0.43	4.97	˂.001*
Sympatry, fw	−1.48 ± 0.45	−3.28	.001*
Sympatry, wf	−1.09 ± 0.45	−2.40	.016*
Low food	−0.77 ± 0.39	−1.97	.049*
Family SMR (high food)	3.51 ± 8.34	0.42	.67
Family SMR: food (low food)*	−14.52 ± 6.55	−2.22	.03*
Family embryo mass	79.57 ± 22.67	3.51	<.001*
Hybrid with farmed mother (fw)			
Intercept (high food)	1.78± 0.41	4.31	<.001*
Low food	−1.35 ± 0.58	−2.33	.02*
Hybrid with wild mother (wf)			
Intercept (high food)	1.65 ± 0.52	3.18	.002*
Low food	−1.63 ± 0.80	−2.03	.04*

* Family SMR (low food): estimate = −11.01, *SE* = 8.42, *Z* = −1.31, *p* = 0.19.

The table and footnote should appear as printed below:
