# Supplementary material for: Can variation in standard metabolic rate explain context‐dependent performance of farmed Atlantic salmon offspring?
Source: Ecol Evol. 2018 Dec 26;9(1):212–22. doi: 10.1002/ece3.4716 (PMC6342125; doi:10.1002/ece3.4716)
Supplement: Supplementary file 1 [file ECE3-9-212-s001.docx]

**Supporting information**

**Can variation in standard metabolic rate explain context-dependent performance of farmed salmon offspring?**

SuppInfo Table 1. Number of mature Atlantic salmon of each sex used to make the Surna-AquaGen and Imsa-AquaGen crosses, and their mean (SD) body mass and length. Also given is the mean (SD) embryo (eyed egg) mass.

| Population | Sex | n | Body mass (kg) | Body length (cm) | Embryo mass (g) |
| --- | --- | --- | --- | --- | --- |
| *Surna-AquaGen* |  |  |  |  |  |
| Surna | Female | 10 | 4.2 (0.8) | 79.5 (4.3) | 0.120 (0.028) |
|  | Male | 10 | 3.1 (1.3) | 70.2 (9.0) |  |
|  |  |  |  |  |  |
| AquaGen | Female | 10 | 12.6 (1.0) | 104.6 (3.1) | 0.141 (0.010) |
|  | Male | 10 | 9.7 (1.0) | 101.9 (2.7) |  |
|  |  |  |  |  |  |
| *Imsa-AquaGen* |  |  |  |  |  |
| Imsa | Female | 11 | 3.0 (1.1) | 63.0 (21.6) | 0.102 (0.015) |
|  | Male | 11 | 1.8 (0.6) | 63.5 (6.4) |  |
|  |  |  |  |  |  |
| AquaGen | Female | 11 | 12.1 (1.2) | 103.5 (2.8) | 0.152 (0.015) |
|  | Male | 11 | 8.6 (0.7) | 94.2 (4.0) |  |
|  |  |  |  |  |  |

SuppInfo Table 2. Number of semi-natural channels stocked with wild and/or farmed-wild hybrid Atlantic salmon juveniles at low or high food availability, and in allopatry or sympatry. Given in parentheses are the number of semi-natural streams for each treatment remaining after omitting streams with holes in the mesh. The number of families and individuals per family used per channel in the different treatments is also given together with a proxy for initial biomass (based on mean female embryo mass) in the stream channels with different competition treatments.

| Types | High food | | Low food | No fam | No ind fam^-1^ | Initial biomass (g) |
| --- | --- | --- | --- | --- | --- | --- |
| *Allopatry* | |  |  |  |  |  |
| Wild | | 4 | 4 (2) | 6 | 6 | 2.59 |
| Hybrids, wild mother | | 4 | 4 (3) | 6 | 6 | 2.59 |
| Hybrids, farmed mother | | 4 | 4 (3) | 6 | 6 | 5.69 |
| *Sympatry* | |  |  |  |  |  |
| Wild and hybrids, wild mother | | 4 | 4 (2) | 12 | 3 | 2.59 |
| Wild and hybrids, farmed mother | | 4 | 4 (2) | 12 | 3 | 4.14 |


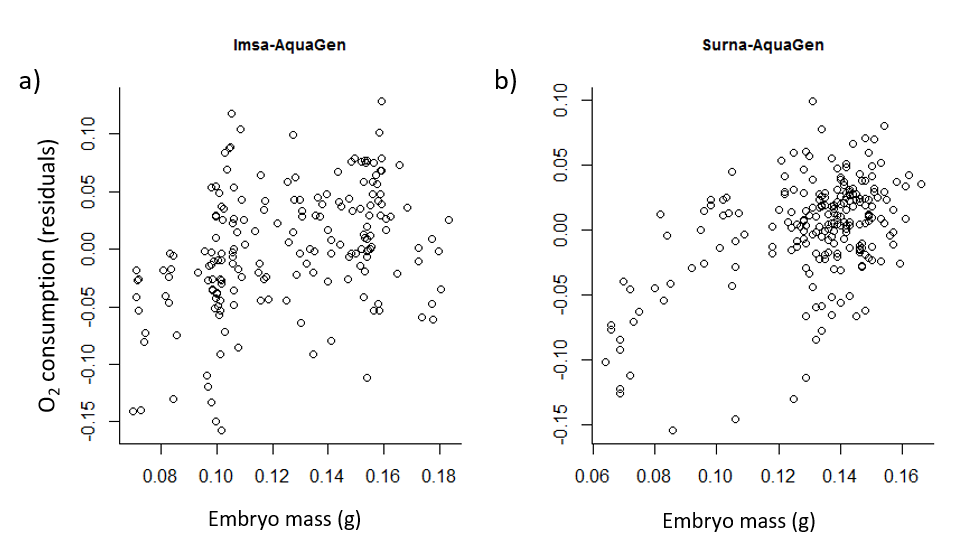


SuppInfo Figure 1. Residual SMR of Atlantic salmon embryos from the a) Imsa-AquaGen and b) Surna-AquaGen crosses, extracted from linear models estimating O2 consumption (log10 transformed) as a function of the duration of the measurement period, plotted on embryo mass. Note that the residuals are not corrected for embryo mass and thus differ from the residuals used as estimates for mass-specific SMR.
